# Hospitalization and Mortality in Patients With Heart Failure Treated With Sacubitril/Valsartan vs. Enalapril: A Real-World, Population-Based Study

**DOI:** 10.3389/fcvm.2020.602363

**Published:** 2021-01-20

**Authors:** Swathi Pathadka, Vincent K. C. Yan, Xue Li, Gary Tse, Eric Y. F. Wan, Hayden Lau, Wallis C. Y. Lau, David C. W. Siu, Esther W. Chan, Ian C. K. Wong

**Affiliations:** ^1^Department of Pharmacology and Pharmacy, Li Ka Shing Faculty of Medicine, Centre for Safe Medication Practice and Research, University of Hong Kong, Hong Kong, China; ^2^Department of Paediatrics and Adolescent Medicine, Li Ka Shing Faculty of Medicine, University of Hong Kong, Hong Kong, China; ^3^Department of Social Work and Social Administration, Faculty of Social Science, University of Hong Kong, Hong Kong, China; ^4^Xiamen Cardiovascular Hospital, Xiamen University, Xiamen, China; ^5^Department of Family Medicine and Primary Care, Li Ka Shing Faculty of Medicine, The University of Hong Kong, Hong Kong, China; ^6^Department of Accident & Emergency, Li Ka Shing Faculty of Medicine, Queen Mary Hospital, University of Hong Kong, Hong Kong, China; ^7^Research Department of Practice and Policy, University College of London School of Pharmacy, London, United Kingdom; ^8^Department of Medicine, Li Ka Shing Faculty of Medicine, The University of Hong Kong, Hong Kong, China

**Keywords:** heart failure, sacubitril/valsartan, enalapril, pharmacoepidemiolgy, mortality, hospitalization

## Abstract

**Background:** The effect of sacubitril/valsartan on survival and hospitalization risk in older patients with heart failure has not been explored. We aimed to investigate the risk of hospitalization and mortality with the use of sacubitril/valsartan vs. enalapril in patients with heart failure.

**Methods:** This was a population-based cohort study using the Hong Kong-wide electronic healthcare database. Patients diagnosed with heart failure and newly prescribed sacubitril/valsartan or enalapril between July 2016 and June 2019 were included. The risk of primary composite outcome of cardiovascular mortality or heart failure-related hospitalization, all-cause hospitalization, heart failure-related hospitalization, cardiovascular mortality and all-cause mortality were compared using Cox regression with inverse probability treatment weighting. Additional analysis was conducted by age stratification.

**Results:** Of the 44,503 patients who received sacubitril/valsartan or enalapril, 3,237 new users (sacubitril/valsartan, *n* = 1,056; enalapril, *n* = 2,181) with a diagnosis of heart failure were identified. Compared with enalapril, sacubitril/valsartan users were associated with a lower risk of primary composite outcome [hazard ratio (HR) 0.58; 95% confidence interval (CI), 0.45–0.75], heart failure-related hospitalization (HR 0.59; 95% CI, 0.45–0.77), all-cause mortality (HR 0.51; 95% CI, 0.36–0.74) and borderline non-significant reductions in all-cause hospitalization (HR 0.85; 95% CI, 0.70–1.04) and cardiovascular mortality (HR 0.63; 95% CI, 0.39–1.02). The treatment effect of sacubitril/valsartan remains unaltered in the patient subgroup age ≥ 65 years (73%).

**Conclusions:** In real-world settings, sacubitril/valsartan was associated with improved survival and reduced heart failure-related hospitalization compared to enalapril in Asian patients with heart failure. The effectiveness remains consistent in the older population.

## Introduction

Sacubitril/valsartan, the first-in-class angiotensin receptor neprilysin inhibitor (ARNI), was proven to be superior to angiotensin-converting enzyme inhibitor (ACEI), with significant reduction in all-cause mortality and composite outcome of heart failure hospitalization or cardiovascular mortality, in patients with heart failure with reduced ejection fraction (HFrEF) in the Prospective Comparison of ARNI With ACEI to Determine Impact on Global Mortality and Morbidity in Heart Failure (PARADIGM-HF) trial (1). The novel drug combination also reduced the rates of 30-day heart failure readmission and all-cause readmission after heart failure hospitalization (2). Based on treatment benefits observed in the pivotal trial, sacubitril/valsartan was indicated for patients with symptomatic HFrEF (3, 4).

Despite compelling evidence on the reduction of mortality and heart failure hospitalization observed in ARNI group in the pivotal trial, the generalisability of findings in clinical practice is uncertain due to the stringent trial criteria in patient recruitment. Approximately 76% of real-world patients with HFrEF did not meet the trial criteria owing to the difference in clinical characteristics and only a quarter of patients in clinics were eligible to receive sacubitril/valsartan (5, 6). Moreover, women, elderly patients and ethnic minorities are usually under-represented in clinical trials involving heart failure, including PARADIGM-HF trial (7). Only 22% of the trial population were women (1), 18% were from the Asia-Pacific region (8), 49% of patients were >65 years (9), and the mean age of death was 65.5 years (10).

Previous retrospective observational studies conducted in the United States (11–13) and Canada (14) reported a reduction in hospitalization and improvement in the quality of life in the Western population, favoring sacubitril/valsartan treatment over ACEI or angiotensin-II receptor blocker (ARB). However, the real-world evidence on the effectiveness of sacubitril/valsartan in the Asian population is limited. Asian patients with heart failure may be younger, but with more cardiovascular mortality and heart failure hospitalizations compared to Western counterparts (9, 15). A subgroup analysis of PARADIGM-HF reported higher cardiovascular and all-cause mortality in patients from the Asia-Pacific region than other regions (8). Notably, there is considerable variability in regional, ethnic, socioeconomic, and aetiological factors within Asia-Pacific countries, which contribute to differences in heart failure characteristics and outcomes (8, 16, 17).

To bridge this research gap, a population-based cohort study using territory-wide electronic medical records (EMR) was conducted to evaluate the impact of sacubitril/valsartan on mortality and hospitalization in Chinese patients with heart failure in the real-world setting of Hong Kong.

## Materials and Methods

### Data Source

This study was conducted using EMR from the Clinical Data Analysis and Reporting System (CDARS) database developed by the Hong Kong Hospital Authority (HA). The HA provides healthcare services to more than 7.4 million people in Hong Kong via 43 public hospitals, 49 specialist outpatient clinics and 73 general outpatient clinics, and contributes to 70% of the Hong Kong healthcare sector (18). CDARS is a centralized information system of medical data of de-identified patients including demographics, diagnoses, dispensing records, laboratory tests, and consultation records. A high degree of coding accuracy has been demonstrated in previous studies for identification of several cardiovascular outcomes such as atrial fibrillation, stroke, and myocardial infarction (19, 20). CDARS has also been used to conduct a number of post-marketing surveillance studies (21).

### Study Design and Cohort Selection

**Figure 1:**
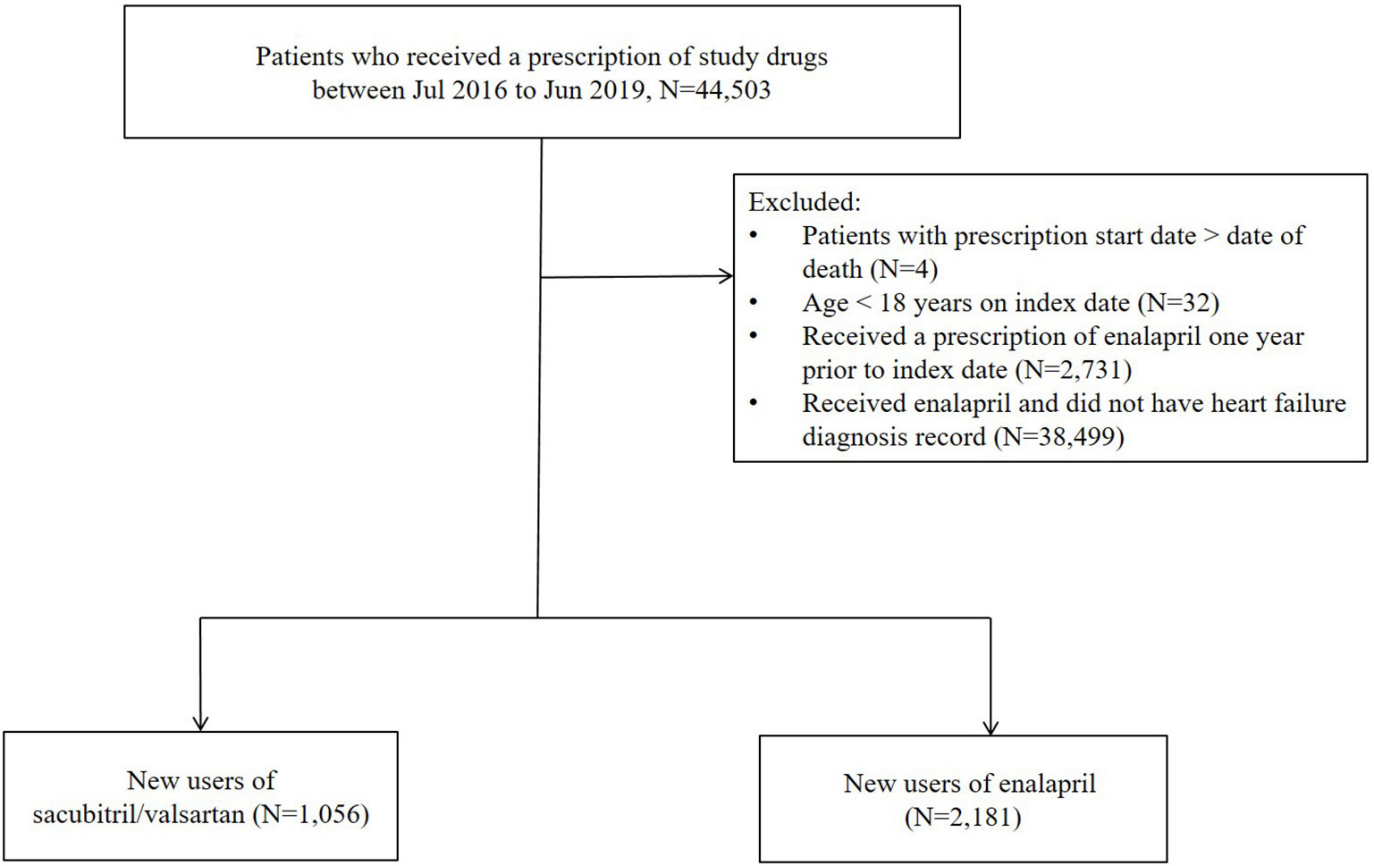
Cohort identification.

This was a retrospective cohort study using a Hong Kong-wide population database. Patients ≥18 years with a clinical diagnosis of heart failure and a prescription of sacubitril/valsartan or enalapril between 1 July 2016 and 30 June 2019 were included. The treatment group was assigned based on the first prescription of drug received during the study period. Patients who received a prescription of enalapril 1 year prior to the index date were excluded to identify new users (Figure 1). Follow-up began from the start date of first prescription of sacubitril/valsartan or enalapril (i.e., index date) until the earliest of censoring events, including the occurrence of an outcome, death, switch in therapy, discontinuation of treatment (since cardiovascular drugs are usually dispensed for 1 month, treatment break of >30 days between consecutive prescriptions), or the end of the study period (31 July 2019).

### Outcomes

The primary outcome was a composite of death from cardiovascular causes or heart failure-related hospitalization. Secondary outcomes were death from any cause, death from cardiovascular cause, all-cause hospitalization, and heart failure-related hospitalization. The outcome of heart failure and cause of death were identified using the International Classification of Diseases (ICD)-9 and ICD-10 codes, respectively (Supplementary Table 1).

## Statistical Analysis

### Primary Analysis

Baseline patient characteristics were expressed as mean [standard deviation (SD)] for continuous variables and as frequencies (percentages) for categorical variables. To minimize the imbalance arising from confounding between sacubitril/valsartan and enalapril treatment groups, inverse probability treatment weighting (IPTW) using propensity score (PS) was employed. PS was estimated using logistic regression with the treatment group as dependent variable and all confounders as independent variables (22). Potential confounders included age, sex, prior history of diseases (hypertension, diabetes mellitus, ischaemic cardiomyopathy, atrial fibrillation, myocardial infarction, ischaemic heart disease, and ischaemic stroke), Charlson comorbidity index, the number of hospitalization (1 year prior to index date), and the number of past heart failure-related hospitalizations (left-censored to the earliest record in CDARS), recent medication use (prescriptions 1 year prior to index date, including renin-angiotensin-aldosterone system inhibitors, beta-blockers, calcium channel blockers, diuretics, digoxin, antithrombotic therapy, anti-arrhythmic therapy, hypoglycaemic drugs, lipid-lowering drugs, mineralocorticoid antagonists). A standardized mean difference of <0.2 between the treatment groups post-weighting was considered negligible (23).

We used the Kaplan Meier curve to illustrate the clinical outcomes between treatment groups. The risk of hospitalization and death was compared between treatment groups using Cox regression. Since the majority of heart failure burden occurs in those aged above 65 years, we conducted subgroup analysis to investigate the comparative effectiveness stratified by age groups (<65 and ≥65 years). To assess the robustness of results of the primary analysis, sensitivity analyses were conducted using 1:1 PS matching with nearest neighbor method (24). and regression adjustment. The statistical analyses were performed using R 3.6.1 (RStudio, Boston, Massachusetts), and independent crosscheck of analysis was conducted by two co-authors (S.P and V.K.C.Y) for quality assurance.

## Results

### Patient Characteristics

A total of 44,503 patients received a prescription of sacubitril/valsartan or enalapril between 1 July 2016 and 31 June 2019 Figure 1. Following the exclusion criteria, 2,181 patients on enalapril and 1,056 patients on sacubitril/valsartan were included in the analysis [age, mean (± SD): 74.2 ± 14.6 years; female: 44.2%; Table 1]. Before weighting, patients on enalapril had a higher burden of chronic cardiovascular diseases, except for ischaemic heart disease. However, the proportion of medication use was lower in sacubitril/valsartan users in general. After weighting, the treatment groups in comparison were well-balanced for all the baseline characteristics.

**Table 1:** Baseline characteristics of cohort adjusted using inverse probability treatment weighting.

	**Enalapril**	**Sacubitril/valsartan**	**SMD**	
	**2,181**	**1,056**	**Crude**	**Adjusted**
Age, mean (SD)	78.25 (13.25)	65.84 (13.82)	0.92	0.07
Sex- female, no. (%)	1,067 (48.9)	307 (29.1)	0.42	0.07
**Comorbidities - no. (%)**				
Atrial fibrillation	717 (32.9)	290 (27.5)	0.12	0.04
Diabetes mellitus	654 (30.0)	265 (25.1)	0.11	0.02
Hypertension	1,163 (53.3)	384 (36.4)	0.35	0.05
Ischaemic cardiomyopathy	17 (0.8)	58 (5.5)	0.27	<0.001
Ischaemic heart disease	777 (35.6)	549 (52.0)	0.33	0.09
Ischaemic stroke	327 (15.0)	87 (8.2)	0.21	0.09
**Recent medication use - no. (%)**				
Anti-arrhythmic drugs	127 (5.8)	157 (14.9)	0.30	0.13
Anti-thrombotic therapy	1,279 (58.6)	875 (82.9)	0.55	0.11
Beta-blockers	711 (32.6)	835 (79.1)	1.06	0.17
Calcium channel blockers	953 (43.7)	254 (24.1)	0.42	0.07
Digoxin	285 (13.1)	200 (18.9)	0.16	0.012
Diuretics	1,225 (56.2)	868 (82.2)	0.59	0.09
Hypoglycaemic drugs	524 (24.0)	342 (32.4)	0.19	0.07
Lipid lowering drugs	845 (38.7)	722 (68.4)	0.62	0.10
Mineralocorticoid antagonists	119 (5.5)	520 (49.2)	1.13	0.07
RAAS inhibitors	658 (30.2)	852 (80.7)	1.18	0.03
Number of hospitalizations 1 year before index date, mean (SD)	2.74 (5.41)	2.73 (6.48)	0.002	0.019
CCI, mean (SD)	2.44 (1.95)	1.92 (1.76)	0.28	0.06
Number of HF hospitalizations before index date, mean (SD)	2.73 (4.31)	2.68 (4.17)	0.01	0.05

*CI, Charlson comorbidity index; HF, heart failure; RAAS inhibitors, renin-angiotensin-aldosterone system inhibitor; SD, standard deviation*.

### Clinical Outcomes of Sacubitril/Valsartan Compared to Enalapril

**Figure 2:**
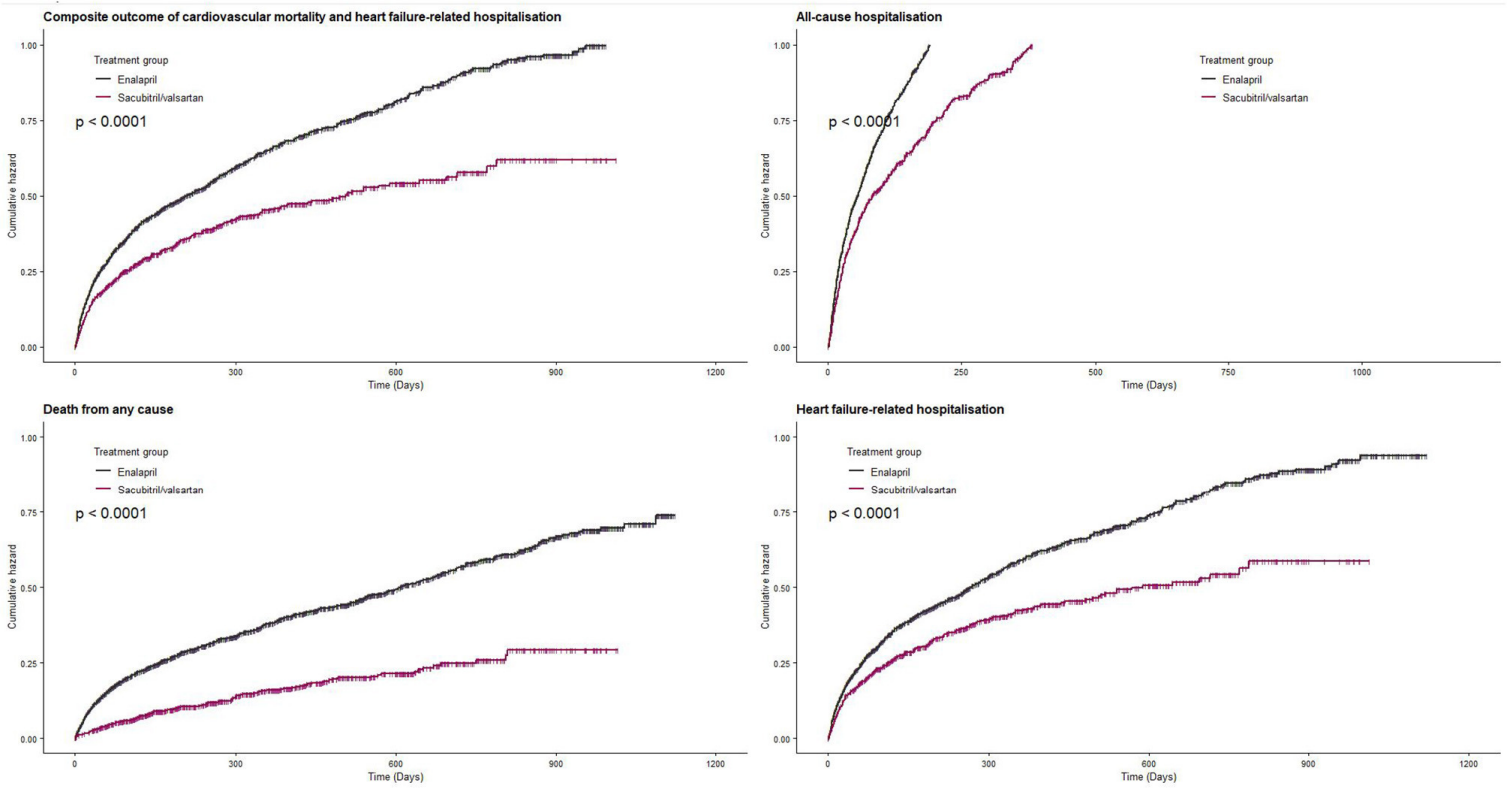
Effect of sacubitril/valsartan on hospitalization and survival in patients with heart failure compared to enalapril.

The primary outcome composite of death from cardiovascular causes or hospitalization due to heart failure-related causes occurred in 32.6% of sacubitril/valsartan group and 47.3% in enalapril group during the median follow-up of 5.3 months (interquartile range: 1.43–14.6). Sacubitril/valsartan use was associated with lower risk of composite outcome compared with enalapril (sacubitril/valsartan vs. enalapril: 46.7 vs. 60.9 per 100 person-years; HR, 0.58; 95% CI, 0.45–0.75) (Figure 2 and Table 2). Compared with enalapril, sacubitril/valsartan use was associated with lower risk of all-cause mortality (HR, 0.51; 95% CI, 0.36–0.74) and heart failure-related hospitalization (HR, 0.59; 95% CI, 0.45–0.77) (Table 2). There was a trend toward lower risk of all-cause hospitalization (HR, 0.85; 95% CI, 0.70–1.04) and cardiovascular mortality (HR, 0.63; 95% CI, 0.39–1.02) in sacubitril/valsartan group, but this was not statistically significant.

**Table 2:** Effect of sacubitril/valsartan on patients with heart failure compared to enalapril (adjusted using inverse probability treatment weighting).

**Outcomes**	**Enalapril**	**Sacubitril/valsartan**	**Crude HR (95% CI)**	**Adjusted HR (95% CI)**
	***N* = 2,181 – no. (%)**	***N* = 1,056 – no. (%)**		
Composite outcome of cardiovascular mortality or heart failure-related hospitalization	1,031 (47.3)	344 (32.6)	0.68 (0.60–0.77)	0.58 (0.45–0.75)
All-cause hospitalization	1,578 (72.3)	599 (56.7)	0.72 (0.66–0.80)	0.85 (0.70–1.04)
Heart failure-related hospitalization	926 (42.5)	320 (30.3)	0.71 (0.62–0.81)	0.59 (0.45–0.77)
All-cause mortality	788 (36.1)	141 (13.4)	0.40 (0.33–0.48)	0.51 (0.36–0.74)
Cardiovascular mortality	214 (9.81)	64 (6.06)	0.65 (0.49–0.86)	0.63 (0.39–1.02)

*CI, confidence interval; HR, hazard ratio*.

### Subgroup Analyses

Approximately 73% of patients included in the cohort were 65 years and above. The beneficial effects of sacubitril/valsartan remained consistent in older age with lower risk of composite outcome (HR, 0.58; 95% CI 0.42–0.85), death from any cause (HR, 0.53; 95% CI, 0.35–0.80), and heart failure-related hospitalization (HR, 0.60; 95% CI, 0.43–0.84) (Table 3). There was no statistically significant difference in the risk of cardiovascular mortality (HR, 0.63; 95% CI, 0.37–1.08) or all-cause hospitalization (HR, 0.85; 95% CI, 0.67–1.08) between the treatment groups.

**Table 3:** Subgroup analysis by age (adjusted using inverse probability treatment weighting).

	**Age** **≥** **65 years**			**Age** **<** **65 years**		
**Outcomes**	**Enalapril**	**Sacubitril/valsartan**	**HR (95% CI)**	**Enalapril**	**Sacubitril/valsartan**	**HR (95% CI)**
	***N* = 1,833 – no. (%)**	***N* = 534 – no. (%)**		***N* = 522 – no. (%)**	***N* = 348 – no. (%)**	
Composite outcome of cardiovascular mortality or heart failure-related hospitalization	885 (48.3)	187 (35.0)	0.58 (0.42–0.85)	146 (42.0)	157 (30.1)	0.59 (0.41–0.85)
All-cause hospitalization	1,345 (73.4)	311 (58.2)	0.85 (0.67–1.08)	233 (66.9)	288 (55.2)	0.87 (0.64–1.18)
Heart failure-related hospitalization	788 (43.0)	171 (32.0)	0.60 (0.43–0.84)	138 (39.7)	149 (28.5)	0.58 (0.40–0.84)
All-cause mortality	740 (40.4)	102 (19.1)	0.53 (0.35–0.80)	48 (13.8)	39 (7.5)	0.47 (0.26–0.87)
Cardiovascular mortality	198 (10.8)	45 (8.4)	0.63 (0.37–1.08)	16 (4.6)	19 (3.6)	0.69 (0.24–2.01)

*CI, confidence interval; HR, hazard ratio*.

### Sensitivity Analyses

Of the 3,237 patients in the study cohort, 503 patients from each treatment group were successfully matched (Supplementary Table 2). The trend of lower risk for all outcomes did not change with PS matching or unweighted multivariable Cox regression adjustment, with minimal difference in treatment effect (Table 4).

**Table 4:** Sensitivity analyses.

**Outcome**	**Multivariable Cox regression**	**After propensity score matching**		
	Adjusted HR* (95% CI)	**Enalapril**	**Sacubitril/valsartan**	**HR**
		***N* = 503 – no. (%)**	***N* = 503– no. (%)**	**(95% CI)**
Composite outcome of death from cardiovascular cause or hospitalization due to heart failure	0.59 (0.49–0.70)	243 (48.3)	149 (29.6)	0.56 (0.45–0.68)
All-cause hospitalization	0.73 (0.64–0.84)	360 (31.6)	285 (56.7)	0.71 (0.60–0.82)
Heart failure-related hospitalization	0.60 (0.50–0.72)	223 (44.3)	134 (26.6)	0.55 (0.44–0.68)
All-cause mortality	0.44 (0.34–0.55)	163 (32.4)	72 (14.3)	0.48 (0.36–0.64)
Cardiovascular mortality	0.58 (0.39–0.86)	48 (9.5)	32 (6.4)	0.69 (0.44–1.08)

* *Adjusted for age, sex, comorbidities, Charlson comorbidity index, number of hospitalizations (any cause or heart failure-related), and recent use of medications; CI, confidence interval; HR, hazard ratio*.

## Discussion

### Study Findings and Comparison With Existing Evidence

This study demonstrates a significant reduction in all-cause mortality and heart failure-related hospitalization with sacubitril/valsartan use in Chinese patients with heart failure compared with enalapril. The results remain consistent to the elderly patient group.

In this population-based, comparative effectiveness study using territory-wide EMR, the patient population had a higher proportion of women, older patients and comorbidities compared to the PARADIGM-HF trial. Given the stringent trial criteria, real-world evidence is important and complements the findings of the randomized controlled trials. In the current literature, patients on sacubitril/valsartan were slightly younger with fewer comorbidities and better health status compared with enalapril users, which is congruent with previous retrospective observational studies in the Western populations (12, 25). However, in our study, the proportion of recent medication used for chronic illness control was higher in the sacubitril/valsartan group. Since sacubitril/valsartan is indicated for stage II-IV patients with HFrEF who remain symptomatic despite treatment with other standard heart failure therapy, the majority of patients are likely to have higher medication use in the sacubitril/valsartan group.

In the PARADIGM-HF trial, sacubitril/valsartan reduced the risk of composite outcome of cardiovascular mortality or heart failure hospitalization by 20% compared with enalapril (1). A recent cohort study involving 7,893 patients with HFrEF in the US also demonstrated reduction in death and hospitalization with sacubitril/valsartan use compared to ACEI/ARB (12). However, the Asian population was under-represented in both studies, accounting for only 18% of the trial population (9) and only 2% of the retrospective cohort study (12). Notably, the treatment effect of sacubitril/valsartan did not reach statistical significance for the primary outcome (HR, 1.39; 95% CI, 1.06–1.80) for the Asia-Pacific region (9). Further, there are noticeable differences in the patient characteristics between ethnicities regarding HFrEF (17). In the real-world setting, eligibility for the medication may differ. For example, 24% of the HFrEF cohort was eligible to receive ARNI in the Swedish registry (6).

To date, one cohort study from Taiwan (*N* = 932) was conducted in Asia, in which sacubitril/valsartan plus standard care was associated with 34% reduction of the composite outcome compared to the standard care without sacubitril/valsartan (26). However, the outcomes were not adjusted for any potential confounding factors, neither using traditional regression nor PS methods. In our study of more than 3,000 patients who were more than 10 years older than the Taiwanese cohort, we observed an association of risk reduction of 41% for the same composite outcome in sacubitril/valsartan group compared to enalapril. This provides further evidence of effectiveness of sacubitril/valsartan in the management of heart failure in the Asian population.

Hospitalization is a strong predictor of survival and quality of life in patients with heart failure (27). Previous studies also demonstrated cost-effectiveness of sacubitril/valsartan compared to ACEI/ARB, with lower hospitalization rate and healthcare costs (13, 28). Compared to the 44% reduction in the risk of heart failure hospitalizations in US (13), use of sacubitril/valsartan compared with enalapril was associated with similar reduction in the risk of heart failure-related hospitalization in this study. In addition to the beneficial effect on hospitalization due to heart failure, sacubitril/valsartan use is also associated with lower risk of death from any cause compared with enalapril. Therefore, sacubitril/valsartan could offer improved patient outcomes and optimize healthcare resource utilization.

The use of sacubitril/valsartan in the elderly population is less studied (9, 29). The risk of mortality is higher in patients with heart failure >65 years compared to non-elderly patients (30). Patients in our study cohort were almost 12 years older than that of the PARADIGM-HF trial population, with nearly three-quarters over 65 years (1). The older population continued to benefit from the effects of sacubitril/valsartan. Further studies focusing on the safety of sacubitril/valsartan in the older population, in patients with renal dysfunction and potential barriers for the clinical adoption of sacubitril/valsartan should be carried out.

## Limitations

This study has several limitations. Firstly, due to the retrospective nature of the study using electronic medical databases, there is potential for selection bias and unmeasured residual confounding. To minimize this potential prescribing bias arising from confounders between the treatment groups, IPTW was employed. The analysis was repeated by using multivariable Cox regression and PS matching that consistently support the robustness of the primary analysis. Secondly, since echocardiography information was not available from the study dataset, we could not distinguish the subtypes of heart failure. However, since sacubitril/valsartan is currently indicated only for symptomatic HFrEF management, patients with a prescription record of sacubitril/valsartan were taken to have a diagnosis of HFrEF. Future studies are warranted with long-term follow-up informing the safety and effectiveness of sacubitril/valsartan compared to other standard HFrEF treatment.

## Clinical Implications

This study provides evidence to support the effectiveness of sacubitril/valsartan in a cohort comprised of an older (mean age, 74 vs. 63.8 years in the PARADIGM-HF trial), more often women (44 vs. 22%) Asian population with heart failure (1). Despite the observed differences in patient characteristics between trial population and real-world population, sacubitril/valsartan was associated with better clinical outcomes in patients with heart failure compared with enalapril. Hence, our study indicates that benefits observed in PARADIGM-HF trial could be translatable to a representative population of Asian patients with heart failure.

Switching from ACEI or ARB to sacubitril/valsartan in stable HFrEF showed improvements in cardiac structure and function and reduction in healthcare utilization in primary care in recent prospective observational studies (31, 32). The lower risk of heart failure-related hospitalization and all-cause mortality remain consistent in the older population in our study. We recommend the initiation of sacubitril/valsartan in all patients eligible for ARNI with careful safety monitoring. Caution should be exercised in older patients during initiation and dose titration due to potential side effects, including systolic hypotension which may lead to falls.

## Conclusion

In Chinese patients with heart failure, sacubitril/valsartan users showed lower risks of heart failure-related hospitalization and all-cause mortality compared with enalapril users, including patients ≥ 65 years of age. Sacubitril/valsartan should be initiated in patients eligible for ARNI to obtain optimal pharmacological treatment. Further studies investigating the long-term safety and effectiveness of sacubitril/valsartan are needed.

## Data Availability Statement

The datasets generated during and/or analyzed during the current study are not publicly available due to the nature of sensitive electronic healthcare data. Requests to access these datasets should be directed to the corresponding author.

## Ethics Statement

The study was approved by the Institutional Review Board of the University of Hong Kong/Hospital Authority Hong Kong West Cluster (reference number: UW18-680). Since this is a retrospective study using electronic healthcare database, no informed consent was needed.

## Author Contributions

EWC, ICKW, SP, XL, and EYFW conceptualized the study and methodology. EWC and ICKW provided resources and supervised the study. SP collected the data, conducted the formal analysis, and cross-checked by VY. SP and VY wrote the original draft. All authors critically reviewed and commented on all other drafts.

## Conflict of Interest

EWC has received research funding from Bayer, Bristol-Myers Squibb, Pfizer, and Takeda, for work unrelated to this study. ICKW has received research funding outside the submitted work from Amgen, Bayer, Bristol-Myers Squibb, GSK, and Janssen. XL has received a research grant from Janssen. The remaining authors declare that the research was conducted in the absence of any commercial or financial relationships that could be construed as a potential conflict of interest.
