# Supplementary material for: Hospitalization and Mortality in Patients With Heart Failure Treated With Sacubitril/Valsartan vs. Enalapril: A Real-World, Population-Based Study
Source: Front Cardiovasc Med. 2021 Jan 20;7:602363. doi: 10.3389/fcvm.2020.602363 (PMC7855850; doi:10.3389/fcvm.2020.602363)
Supplement: Supplementary file 1 [file Data_Sheet_1.docx]

**Supplementary Table 1: List of ICD-9-CM codes for identification of medical diagnoses**

| **ICD-9-CM codes** | **Descriptions** |  |  |
| --- | --- | --- | --- |
| Atrial fibrillation | |  |  |
| 427.3 | Atrial fibrillation and flutter |  |  |
| 427 | Cardiac dysrhythmias |  |  |
| Myocarditis | |  |  |
| 130.3 | Myocarditis due to toxoplasmosis |  |  |
| 391.2 | Acute rheumatic myocarditis |  |  |
| 398.0 | Rheumatic myocarditis |  |  |
| 422 | Acute myocarditis |  |  |
| 429.0 | Myocarditis, unspecified |  |  |
| 032.82 | Diphtheritic myocarditis |  |  |
| 036.43 | Meningococcal myocarditis |  |  |
| 074.23 | Coxsackie myocarditis |  |  |
| 093.82 | Syphilitic myocarditis |  |  |
| Pulmonary embolism | |  |  |
| 415.1 | Pulmonary embolism and infarction |  |  |
| Ischaemic heart disease | |  |  |
| 410-414 | Ischaemic heart disease |  |  |
| Ischaemic stroke | | |  |
| 433.01 | Occlusion and stenosis of basilar artery with cerebral infarction | |  |
| 433.11 | Occlusion and stenosis of carotid artery with cerebral infarction | |  |
| 433.21 | Occlusion and stenosis of vertebral artery with cerebral infarction | |  |
| 433.31 | Occlusion and stenosis of multiple and bilateral precerebral arteries with cerebral infarction | |  |
| 433.81 | Occlusion and stenosis of other specified precerebral artery with cerebral infarction | |  |
| 433.91 | Occlusion and stenosis of unspecified precerebral artery with cerebral infarction | |  |
| 434 | Occlusion of cerebral arteries | |  |
| 436 | Acute, but ill-defined, cerebrovascular disease | |  |
| 437.0 | Cerebral atherosclerosis | |  |
| 437.1 | Other generalized ischaemic cerebrovascular disease | |  |
| Intracranial haemorrhage | | |  |
| 430 | Subarachnoid haemorrhage | |  |
| 431 | Intracerebral haemorrhage | |  |
| 432 | Other and unspecified intracranial haemorrhage | |  |
| Congestive heart failure | | |  |
| 398.91 | Rheumatic heart failure (congestive) | |  |
| 402.01 | Malignant hypertensive heart disease with heart failure | |  |
| 402.11 | Benign hypertensive heart disease with heart failure | |  |
| 402.91 | Unspecified hypertensive heart disease with heart failure | |  |
| 404.01 | Hypertensive heart and chronic kidney disease, malignant, with heart failure and with chronic kidney disease stage I through stage IV, or unspecified | |  |
| 404.03 | Hypertensive heart and chronic kidney disease, malignant, with heart failure and with chronic kidney disease stage V or end stage renal disease | |  |
| 404.11 | Hypertensive heart and chronic kidney disease, benign, with heart failure and with chronic kidney disease stage I through stage IV, or unspecified | |  |
| 404.13 | Hypertensive heart and chronic kidney disease, benign, with heart failure and chronic kidney disease stage V or end stage renal disease | |  |
| 404.91 | Hypertensive heart and chronic kidney disease, unspecified, with heart failure and with chronic kidney disease stage I through stage IV, or unspecified | |  |
| 404.93 | Hypertensive heart and chronic kidney disease, unspecified, with heart failure and chronic kidney disease stage V or end stage renal disease | |  |
| 428.x | Heart failure | |  |
| Cardiomyopathy | | |  |
| 414.8 | Ischaemic cardiomyopathy | |  |
| 425.2 | Obscure cardiomyopathy of Africa | |  |
| 425.5 | Alcoholic cardiomyopathy | |  |
| 425.7 | Nutritional and metabolic cardiomyopathy | |  |
| 425.8 | Cardiomyopathy in other diseases classified elsewhere | |  |
| Hypertension | | |  |
| 401 | Essential hypertension | |  |
| 402 | Hypertensive heart disease | |  |
| 403 | Hypertensive chronic kidney disease | |  |
| 404 | Hypertensive heart and chronic kidney disease | |  |
| 405 | Secondary hypertension | |  |
| 437.2 | Hypertensive encephalopathy | |  |
| Diabetes |  | |  |
| 250 | Diabetes mellitus | |  |
| Transient ischaemic attack | | |  |
| 435 | Transient cerebral ischemia | |  |
| Vascular disease | | |  |
| 410-414 | Ischaemic heart disease | |  |
| 443.8 | Other specified peripheral vascular diseases | |  |
| 443.9 | Peripheral vascular disease, unspecified | |  |
| Myocardial infarction | | | |
| 410 | Acute myocardial infarction | | |
| 412 | Old myocardial infarction | | |
| Cerebrovascular disease | | | |
| 430-438 | Cerebrovascular disease | | |
| Chronic obstructive pulmonary disease | | | |
| 490-496 | Chronic Obstructive Pulmonary Disease and Allied Conditions | | |
| 500 | Coal workers' pneumoconiosis | | |
| 501 | Asbestosis | | |
| 502 | Pneumoconiosis due to other silica or silicates | | |
| 503 | Pneumoconiosis due to other inorganic dust | | |
| 504 | Pneumonopathy due to inhalation of other dust | | |
| 505 | Pneumoconiosis, unspecified | | |
| 506.4 | Respiratory conditions due to chemical fumes and vapours | | |
| Alcohol use | | | |
| 291 | Alcohol-induced mental disorders | | |
| 303 | Alcohol dependence syndrome | | |
| 305.0 | Nondependent alcohol abuse | | |
| 357.5 | Alcoholic polyneuropathy | | |
| 425.5 | Alcoholic cardiomyopathy | | |
| 535.3 | Alcoholic gastritis | | |
| 571.0 | Alcoholic fatty liver | | |
| 571.1 | Acute alcoholic hepatitis | | |
| 571.2 | Alcoholic cirrhosis of liver | | |
| 571.3 | Alcoholic liver damage, unspecified | | |
| 790.3 | Excessive blood level of alcohol | | |
| 977.3 | Poisoning by alcohol deterrents | | |
| 980 | Toxic effect of alcohol | | |
| V11.3 | Personal history of alcoholism | | |
| Valvular heart surgery (procedure codes) | |  |  |
| 35.20 | Open and other replacement of unspecified heart valve |  |  |
| 35.22 | Open and other replacement of aortic valve |  |  |
| 35.24 | Open and other replacement of mitral valve |  |  |
| 35.26 | Open and other replacement of pulmonary valve |  |  |
| 35.28 | Open and other replacement of tricuspid valve |  |  |
| Transient or secondary atrial fibrillation | |  |  |
| Cardiac surgery (procedure codes) | |  |  |
| 00.5 | Other cardiovascular procedures |  |  |
| 35 | Operations on valves and septa of heart |  |  |
| 36 | Operations on vessels of heart |  |  |
| 37 | Other operations on heart and pericardium |  |  |
| Chest pain (Clinical presentation) | |  |  |
| 786.05 | Chest pain |  |  |
| **Charlson comorbidity index calculation** | |  |  |
| Myocardial infarction |  |  |  |
| 410 | Acute myocardial infarction |  |  |
| 412 | Old myocardial infarction |  |  |
|  |  |  |  |
| Congestive heart failure |  |  |  |
| 398.91 | Rheumatic heart failure (congestive) |  |  |
| 402.01 | Malignant hypertensive heart disease with heart failure |  |  |
| 402.11 | Benign hypertensive heart disease with heart failure |  |  |
| 402.91 | Unspecified hypertensive heart disease with heart failure |  |  |
| 404.01 | Hypertensive heart and chronic kidney disease, malignant, with heart failure and with chronic kidney disease stage I through stage IV, or unspecified |  |  |
| 404.03 | Hypertensive heart and chronic kidney disease, malignant, with heart failure and with chronic kidney disease stage V or end stage renal disease |  |  |
| 404.11 | Hypertensive heart and chronic kidney disease, benign, with heart failure and with chronic kidney disease stage I through stage IV, or unspecified |  |  |
| 404.13 | Hypertensive heart and chronic kidney disease, benign, with heart failure and chronic kidney disease stage V or end stage renal disease |  |  |
| 404.91 | Hypertensive heart and chronic kidney disease, unspecified, with heart failure and with chronic kidney disease stage I through stage IV, or unspecified |  |  |
| 404.93 | Hypertensive heart and chronic kidney disease, unspecified, with heart failure and chronic kidney disease stage V or end stage renal disease |  |  |
| 428 | Heart failure |  |  |
|  |  |  |  |
| Peripheral vascular disease |  |  |  |
| 441 | Aortic aneurysm and dissection |  |  |
| 443.9 | Peripheral vascular disease, unspecified |  |  |
| 785.4 | Gangrene |  |  |
| V43.4 | Blood vessel replaced by other means |  |  |
|  |  |  |  |
|  |  |  |  |
| Cerebrovascular disease |  |  |  |
| 430-438 | Cerebrovascular disease |  |  |
|  |  |  |  |
| Chronic obstructive pulmonary disease |  |  |  |
| 490-496 | Chronic Obstructive Pulmonary Disease and Allied Conditions |  |  |
| 500 | Coal workers' pneumoconiosis |  |  |
| 501 | Asbestosis |  |  |
| 502 | Pneumoconiosis due to other silica or silicates |  |  |
| 503 | Pneumoconiosis due to other inorganic dust |  |  |
| 504 | Pneumonopathy due to inhalation of other dust |  |  |
| 505 | Pneumoconiosis, unspecified |  |  |
| 506.4 | Respiratory conditions due to chemical fumes and vapours |  |  |
|  |  |  |  |
| Dementia |  |  |  |
| 290 | Dementias |  |  |
|  |  |  |  |
| Paralysis |  |  |  |
| 342 | Hemiplegia and hemiparesis |  |  |
| 344.1 | Paraplegia |  |  |
|  |  |  |  |
| Diabetes without chronic complication |  |  |  |
| 250.0 | Diabetes mellitus without mention of complication |  |  |
| 250.1 | Diabetes with ketoacidosis |  |  |
| 250.2 | Diabetes with hyperosmolarity |  |  |
| 250.3 | Diabetes with other coma |  |  |
| 250.7 | Diabetes with peripheral circulatory disorders |  |  |
|  |  |  |  |
| Diabetes with chronic complication |  |  |  |
| 250.4 | Diabetes with renal manifestations |  |  |
| 250.5 | Diabetes with ophthalmic manifestations |  |  |
| 250.6 | Diabetes with neurological manifestations |  |  |
|  |  |  |  |
| Chronic renal failure |  |  |  |
| 582 | Chronic glomerulonephritis |  |  |
| 583.0 | Nephritis and nephropathy, not specified as acute or chronic, with lesion of proliferative glomerulonephritis |  |  |
| 583.1 | Nephritis and nephropathy, not specified as acute or chronic, with lesion of membranous glomerulonephritis |  |  |
| 583.2 | Nephritis and nephropathy, not specified as acute or chronic, with lesion of membranoproliferative glomerulonephritis |  |  |
| 583.4 | Nephritis and nephropathy, not specified as acute or chronic, with lesion of rapidly progressive glomerulonephritis |  |  |
| 583.6 | Nephritis and nephropathy, not specified as acute or chronic, with lesion of renal cortical necrosis |  |  |
| 583.7 | Nephritis and nephropathy, not specified as acute or chronic, with lesion of renal medullary necrosis |  |  |
| 585 | Chronic kidney disease (ckd) |  |  |
| 586 | Renal failure, unspecified |  |  |
| 588 | Disorders resulting from impaired renal function |  |  |
|  |  |  |  |
| Mild liver disease (Various cirrhodites) |  |  |  |
| 571.2 | Alcoholic cirrhosis of liver |  |  |
| 571.4 | Chronic hepatitis |  |  |
| 571.5 | Cirrhosis of liver without mention of alcohol |  |  |
| 571.6 | Biliary cirrhosis |  |  |
|  |  |  |  |
| Moderate-severe liver disease |  |  |  |
| 456.0 | Esophageal varices with bleeding |  |  |
| 456.1 | Esophageal varices without bleeding |  |  |
| 456.2 | Esophageal varices in diseases classified elsewhere |  |  |
| 572.2 | Hepatic encephalopathy |  |  |
| 572.3 | Portal hypertension |  |  |
| 572.4 | Hepatorenal syndrome |  |  |
| 572.8 | Other sequelae of chronic liver disease |  |  |
|  |  |  |  |
| Ulcers |  |  |  |
| 531 | Gastric ulcer |  |  |
| 532 | Duodenal ulcer |  |  |
| 533 | Peptic ulcer site unspecified |  |  |
| 534 | Gastrojejunal ulcer |  |  |
|  |  |  |  |
| Rheumatoid arthritis and other inflammatory polyarthropathies |  |  |  |
| 710.0 | Systemic lupus erythematosus |  |  |
| 710.1 | Systemic sclerosis |  |  |
| 710.4 | Polymyositis |  |  |
| 714.0 | Rheumatoid arthritis |  |  |
| 714.1 | Felty's syndrome |  |  |
| 714.2 | Other rheumatoid arthritis with visceral or systemic involvement |  |  |
| 714.81 | Rheumatoid lung |  |  |
| 725 | Polymyalgia rheumatica |  |  |
|  |  |  |  |
| Acquired immune deficiency syndrome |  |  |  |
| 042 | Human immunodeficiency virus [HIV] disease |  |  |
|  |  |  |  |
| Malignancy |  |  |  |
| 140-149 | Malignant neoplasm of lip, oral cavity, and pharynx |  |  |
| 150-159 | Malignant neoplasm of digestive organs and peritoneum |  |  |
| 160-165 | Malignant neoplasm of respiratory and intrathoracic organs |  |  |
| 170-172, 174-176 | Malignant neoplasm of bone, connective tissue, and breast |  |  |
| 179-189 | Malignant neoplasm of genitourinary organs |  |  |
| 190-195 | Malignant neoplasm of other sites |  |  |
| 200-208 | Malignant neoplasm of lymphatic and hematopoietic tissue |  |  |
|  |  |  |  |
| Metastatic solid tumour |  |  |  |
| 196 | Secondary and unspecified malignant neoplasm of lymph nodes |  |  |
| 197 | Secondary malignant neoplasm of respiratory and digestive systems |  |  |
| 198 | Secondary malignant neoplasm of other specified sites |  |  |
| 199 | Malignant neoplasm without specification of site |  |  |
|  |  |  |  |

**Supplementary Table 2. Baseline characteristics of cohort post-matching**

|  | **Enalapril**  **N=503** | **Sacubitril/valsartan**  **N=503** | **SMD** |
| --- | --- | --- | --- |
| **Age, mean (SD)** | 71.28 (14.99) | 70.37 (13.92) | 0.063 |
| **Sex- female, no. (%)** | 199 (39.6) | 180 (35.8) | 0.078 |
| **Comorbidities - no. (%)** | | | |
| Atrial fibrillation | 149 (29.6) | 150 (29.8) | 0.004 |
| Diabetes mellitus | 129 (25.6) | 141 (28.0) | 0.054 |
| Hypertension | 226 (44.9) | 222 (44.1) | 0.016 |
| Ischaemic cardiomyopathy | 10 ( 2.0) | 22 ( 4.4) | 0.136 |
| Ischaemic heart disease | 231 (45.9) | 237 (47.1) | 0.024 |
| Ischaemic stroke | 54 (10.7) | 47 ( 9.3) | 0.046 |
| Myocardial infarction | 124 (24.7) | 129 (25.6) | 0.023 |
| **Recent medication use - no. (%)** | | | |
| Anti-arrhythmic drugs | 63 (12.5) | 61 (12.1) | 0.012 |
| Anti-thrombotic therapy | 366 (72.8) | 382 (75.9) | 0.073 |
| Beta-blockers | 293 (58.3) | 309 (61.4) | 0.065 |
| Calcium channel blockers | 179 (35.6) | 175 (34.8) | 0.017 |
| Digoxin | 83 (16.5) | 93 (18.5) | 0.052 |
| Diuretics | 352 (70.0) | 368 (73.2) | 0.071 |
| Hypoglycaemic drugs | 149 (29.6) | 158 (31.4) | 0.039 |
| Lipid lowering drugs | 293 (58.3) | 306 (60.8) | 0.053 |
| Mineralocorticoid antagonists | 89 (17.7) | 107 (21.3) | 0.09 |
| RAAS inhibitors | 294 (58.4) | 318 (63.2) | 0.098 |
| **Number of hospitalisations one year before index date, mean (SD)** | 2.96 (5.51) | 2.83 (8.94) | 0.017 |
| **CCI, mean (SD)** | 2.14 (1.63) | 2.07 (1.99) | 0.037 |
| **Number of HF hospitalisations before index date, mean (SD)** | 2.53 (3.22) | 2.66 (4.72) | 0.033 |

Abbreviations: CCI, Charlson comorbidity index; HF, heart failure; RAAS, renin-angiotensin-aldosterone system; SD, standard deviation; SMD, standardized mean difference.
